# A Novel Patient Engagement Platform Using Accessible Text Messages and Calls (Epharmix): Feasibility Study

**DOI:** 10.2196/formative.7211

**Published:** 2017-09-18

**Authors:** Avik Som, Kunjan Patel, Eric Sink, Robert Mattson Peters, Kavon Javaherian, Jacob Groenendyk, Tonya An, Zhuchen Xu, Gregory M Polites, Melvin Blanchard, Will Ross

**Affiliations:** ^1^ Washington University School of Medicine St. Louis, MO United States; ^2^ Saint Louis University School of Medicine St. Louis, MO United States; ^3^ Department of Internal Medicine, Renal Division Washington University School of Medicine St. Louis, MO United States

**Keywords:** telemedicine, mobile health, eHealth, telehealth, mHealth innovations, bioinformatics, multiple chronic conditions

## Abstract

**Background:**

Patient noncompliance with therapy, treatments, and appointments represents a significant barrier to improving health care delivery and reducing the cost of care. One method to improve therapeutic adherence is to improve feedback loops in getting clinically acute events and issues to the relevant clinical providers as necessary (ranging from detecting hypoglycemic events for patients with diabetes to notifying the provider when patients are out of medications). Patients often don’t know which information should prompt a call to their physician and proactive checks by the clinics themselves can be very resource intensive. We hypothesized that a two-way SMS system combined with a platform web service for providers would enable both high patient engagement but also the ability to detect relevant clinical alerts.

**Objective:**

The objectives of this study are to develop a feasible two-way automated SMS/phone call + web service platform for patient-provider communication, and then study the feasibility and acceptability of the Epharmix platform. First, we report utilization rates over the course of the first 18 months of operation including total identified clinically significant events, and second, review results of patient user-satisfaction surveys for interventions for patients with diabetes, COPD, congestive heart failure, hypertension, surgical site infections, and breastfeeding difficulties.

**Methods:**

To test this question, we developed a web service + SMS/phone infrastructure (“Epharmix”). Utilization results were measured based on the total number of text messages or calls sent and received, with percentage engagement defined as a patient responding to a text message at least once in a given week, including the number of clinically significant alerts generated. User satisfaction surveys were sent once per month over the 18 months to measure satisfaction with the system, frequency and degree of communication. Descriptive statistics were used to describe the above information.

**Results:**

In total, 28,386 text messages and 24,017 calls were sent to 929 patients over 9 months. Patients responded to 80% to 90% of messages allowing the system to detect 1164 clinically significant events. Patients reported increased satisfaction and communication with their provider. Epharmix increased the number of patient-provider interactions to over 10 on average in any given month for patients with diabetes, COPD, congestive heart failure, hypertension, surgical site infections, and breastfeeding difficulties.

**Conclusions:**

Engaging high-risk patients remains a difficult process that may be improved through novel, digital health interventions. The Epharmix platform enables increased patient engagement with very low risk to improve clinical outcomes. We demonstrated that engagement among high-risk populations is possible when health care comes conveniently to where they are.

## Introduction

There has been significant interest in the digital health field to use automated and digital health techniques to facilitate scalable, proactive patient outreach. Many attempts have been made using Health Insurance Portability and Accountability Act (HIPAA)-secure application and portal environments to engage patients in their care under encrypted safe harbor provisions as defined by the Health Information Technology for Economic and Clinical Health Act (HITECH) [1-5]. Although these applications may be convenient for some providers and patients, for other patients—particularly those who tend to be nonadherent, high-resource users and Medicaid beneficiaries—application and portal usage can be a significant burden due to lack of Internet access, mobile data costs, downloading mechanics, or time required for implementation [2,6-9].

To do our utmost to help these patients per the provisions of HIPAA, we devised a method that uses widely existing infrastructure to meet patient care needs. Almost all (97%) patients carry a cell phone, and 85% of homeless veterans carry a cell phone [10-12]. All cell phones come with both voice and short message service (SMS) applications preinstalled. All landlines can carry a voice application. Newly developed voice on the cloud technology has allowed us to build an automated software platform that contacts patients en masse with the additional attribute of being toll-free, allowing us to reach low-income patients who may not have minutes or texts available for other purposes. The software platform that our team developed is called Epharmix, and it was implemented at Washington University in St. Louis as a quality improvement initiative.

Although phone calls fall under the safe harbor provisions of the HITECH act, making them automated means that they must meet the requirements put forward by the Federal Communications Commission (FCC) under the Telephone Consumer Protection Act (TCPA) [13-15]. Additionally, due to HIPAA constraints, the content of the messages must be strictly controlled to mitigate the risk of breach of protected health information (PHI) [16,17].

We found that for patients older than 65 years, phone calls were an appropriate and effective means to contact patients. However, for patients under 65 years, text messages are generally seen as a more effective medium for communication. In our system, SMS messages come from toll-free numbers to the patients and on request can be made free to the end user, but SMS messages are intrinsically unencrypted. To mitigate the risk of a reportable breach, we developed a series of measures including scrubbing messages of all PHI, obtaining informed consent, phone line-specific consenting, and using secure servers.

**Figure 1:**
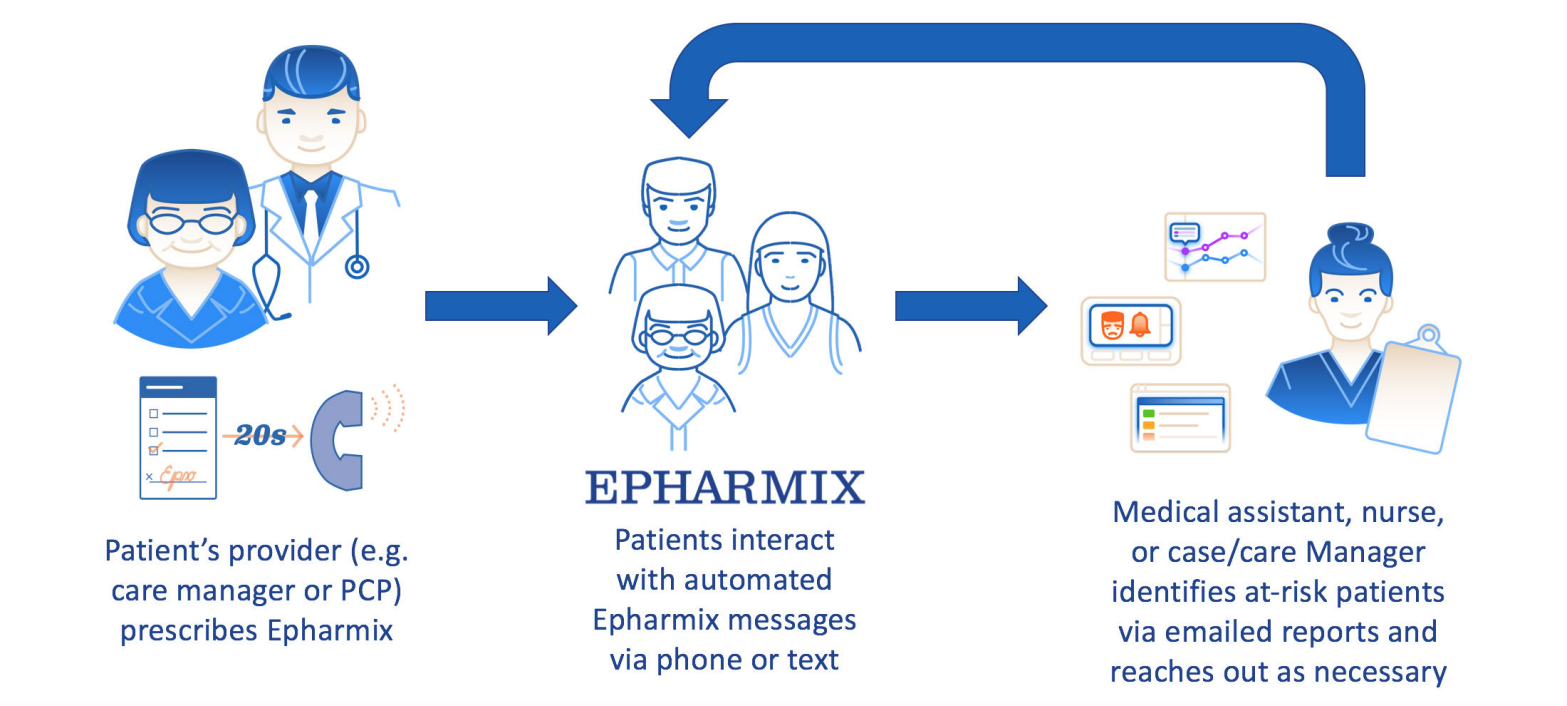
Patient data flow between provider and patient in Epharmix system.

The Epharmix system possesses several key features to maximize data security and protect patient and provider privacy. Figure 1 describes the organization of data flow in the system. A detailed description of individual disease specific algorithms and interventions can be found on www.epharmix.com. These include building a combination of security measures including infrastructure, identifier removal, clinically relevant surveys, clinical data reporting, voluntary opt-out, toll-free messaging, customizable message frequency, consent confirmation, and time tracking, that are all built into the software. In addition, with our clinical partners we built a process infrastructure including a business associate agreement (BAA), patient consent, and provider workflow. Each of these is described more thoroughly below.

We developed the Epharmix platform with both high availability and patient security in mind. The Epharmix stack was deployed on servers provided by an industry-leading, Health Information Trust Alliance–certified hosting provider. The environment was hardened on the operating system level and included features such as around the clock penetration monitoring, distributed denial of service mitigation, intelligent intellectual property reputation filters, and additional security measures. All data held were placed in file encryption vaults using the AES-256 encryption algorithm; the vaults were managed by a role-based access control system to ensure maximum security. Finally, following log-in, a log-out timer automatically logs the user out of the website after it has been idle for over 30 minutes.

Strict control of the messaging was implemented with all messages pre-vetted and any identifiers removed, ensuring that providers could not send identifying information via either SMS or phone call versions. Messages from patients were restricted to numerical values, single letter, or yes/no answers; no free-form text responses were allowed. This further mitigated the risk of the patient disclosing their own PHI.

Working directly with clinicians, we built a library of 20 disease-specific surveys capable of identifying signs and symptoms across various patient populations. These surveys trigger alerts and reports that providers can review in order to improve clinical management. Providers received alerts via email, text message, page, or phone call when urgent data were ready for their review. These alerts contained no PHI but still relayed the pertinent data necessary to maintain clinical usefulness. Providers were able to access identifiable data by logging in to the secure Web-based portal. Data from the patient regarding patient-reported signs and symptoms can be stored in the Epharmix portal or transferred into the appropriate electronic medical record (EMR). A voluntary opt-out of the service was built into Epharmix to respond to a patient replying “STOP” or pressing the asterisk on his or her phone. This feature cancels all future messages and ensures compliance with federal communication commission regulations.

Phone calls are initiated from a toll-free number to remove any fees that may otherwise be charged to the patient, whether from a cell phone or a landline. Additionally, a free-to-end-user service makes all SMS text messages free to the end user through the use of an approved short code messaging sequence within the Epharmix software algorithm. For some patients, standard messaging charges may still apply based on their specific mobile plans, but every effort is made to remove these fees.

Message frequency and timing is controlled by the patient initially. In addition, a smart frequency algorithm can modulate the frequency based on the patient’s self-reported condition, for instance: when a patient reports values within the provider set thresholds, the message frequency is decreased in order to prevent user fatigue. Likewise, the smart system will also increase message frequency if the clinical thresholds are breached (e.g. fasting blood glucose > 400 mg/dL or >2 events of paroxysmal nocturnal dyspnea or fluid weight gain in heart failure, among others).

To initiate an Epharmix outward message, providers must confirm that they consented the patient. Then, Epharmix confirms the patient’s identity and informed consent by asking the first name without including any other identifiers or health information. If a patient does not confirm their acceptance to begin receiving messages, the prescribing provider is notified and the patient is sent no further messages.

Finally, as an added feature, Epharmix tracks all time spent by provider and patient on the phone when using the Epharmix system. The time providers spend managing their patients over the phone can be submitted for reimbursement through newly created chronic care management billing codes. Epharmix creates patient time logs that track the amount of time spent in communication with each patient, which can be used as proof of service when filing for reimbursement.

To implement in the clinic a series of process innovations also needed to be made, including developing a business associate agreement between Epharmix, Inc. and its healthcare implementation sites to allow for data sharing and to define data ownership, as well as defining how patients should consent and how providers would access the data.

Per the implementation site policy, a written authorization form which relayed the risks of unencrypted messaging was required to begin sending SMS messages to patients. For patients not using SMS messages, verbal consent to reach them via automated phone calls was sufficient to begin messages.

Providing reliable and timely reports to providers was a central focus of the Epharmix design. Assistance to providers in viewing their patients’ Epharmix data was accomplished through two key methods. The Epharmix software tracks if a provider called their patient or if an alert has already been resolved. For many disease systems, patients are triaged into red, yellow, and green categories based on their self-reported disease severity. For example, patients with diabetes are sorted according to a moving average of their blood glucose values in the past week. This triage system allows providers to focus their calls on the patients in greatest need of their attention at any time.

At 18 months after system implementation, patients using Epharmix demonstrate a 2- to 3-fold increase in response rate compared to general EMR portal usage [18-20]. The results demonstrate a basal weekly response rate of approximately 80% to both text and phone call messages, with up to a 95% response rate depending on the message frequency and particular disease focus. Additionally, Epharmix messages are already being used for the early detection and intervention of clinically significant events such as chronic obstructive pulmonary disease (COPD) exacerbations, heart failure decompensation, and hypoglycemia.

## Methods

### Overview

Epharmix was developed as an iterative process of design and feedback within the Sling Health (formerly IDEA Labs) innovation incubator at Washington University in St. Louis [21]. A combination of software and process development was used to build a system in compliance with all current healthcare, privacy, and communication regulations. The data reported herein is an aggregate analysis of de-identified data from enrollment in quality improvement projects and randomized controlled trials utilizing the Epharmix system currently underway that was reviewed and approved by the Washington University in St. Louis Institutional Review Board. Three of these studies are currently in press or published [22-26]. Individual analysis of disease specific outcomes and interventions is reported separately. This study reports on the engineering and development of the platform as a whole and its subsequent utilization and feasibility for use in the clinic.

### Study Design: Patient Engagement and Satisfaction Analysis

All patients who used Epharmix also received a patient satisfaction survey once per month via text message as part of the implementation. Aggregate deidentified data of the use of Epharmix at Washington University was reported from Epharmix and used in this analysis. No patient specific records were reviewed in this analysis. Because of the aggregate nature of the data across multiple specialties, socioeconomic data could not be collected or analyzed for this particular utilization study. The majority of patients were all adults from St. Louis City and County, and census data for socioeconomic status is reported as a corollary in Table 1 (US Census).

### Measures

Number of text messages/phone calls sent and received is defined as the number successfully sent and received from Epharmix. Patients were defined as “not engaged weekly” if they responded 0 times, and they were defined as “engaged weekly” if they responded 1 or more times in a week. The weekly percentage engagement was then the percentage of patients who were “engaged weekly” in any given week as defined above. Similarly, we also calculated the percentage of patients who were engaged monthly by defining “engaged monthly” as responding to 1 or more messages in a month. And then the monthly percentage engagement was then the percentage of patients who were “engaged monthly” in any given week as defined above. Alerts are defined per disease specific intervention and are considered clinically significant if a patient’s response is above or below clinically set thresholds, such as a blood glucose > 400. Patient satisfaction with their provider using the service was defined based on a Likert scale from 0-9, with 0 being the worst and 9 being the best. Message frequency was also assessed on a 0 to 9 scale, albeit with 5 being perfect, 0 being too few, and 9 being too many. Finally, patients also report the degree to which the system improved communication with their provider on a 0 to 9 basis where 0 is greatly reduced, 5 is stayed the same, and 9 is greatly improved.

### Analysis

Data on engagement and use were analyzed after 18 months of use across a series of disease-specific quality improvement projects and RCTs aimed at improving disease-specific outcomes. Engagement to the Epharmix system reported herein was compared to engagement in the literature seen with health care portals using descriptive statistics.

**Table 1:** St. Louis city and county residents demographic and income data from which the Epharmix population was recruited (US Census).

Characteristics	St. Louis city	St. Louis county
Age 18-65 years, %	61.2	55.2
Age >65 years, %	11	16.8
Male, %	48.3	47.7
Female, %	51.7	52.3
White, %	43.9	69.5
African American, %	49.2	24.1
Median household income 2011-2015	$35,599	$59,755

## Results

### Software Development

**Figure 2:**
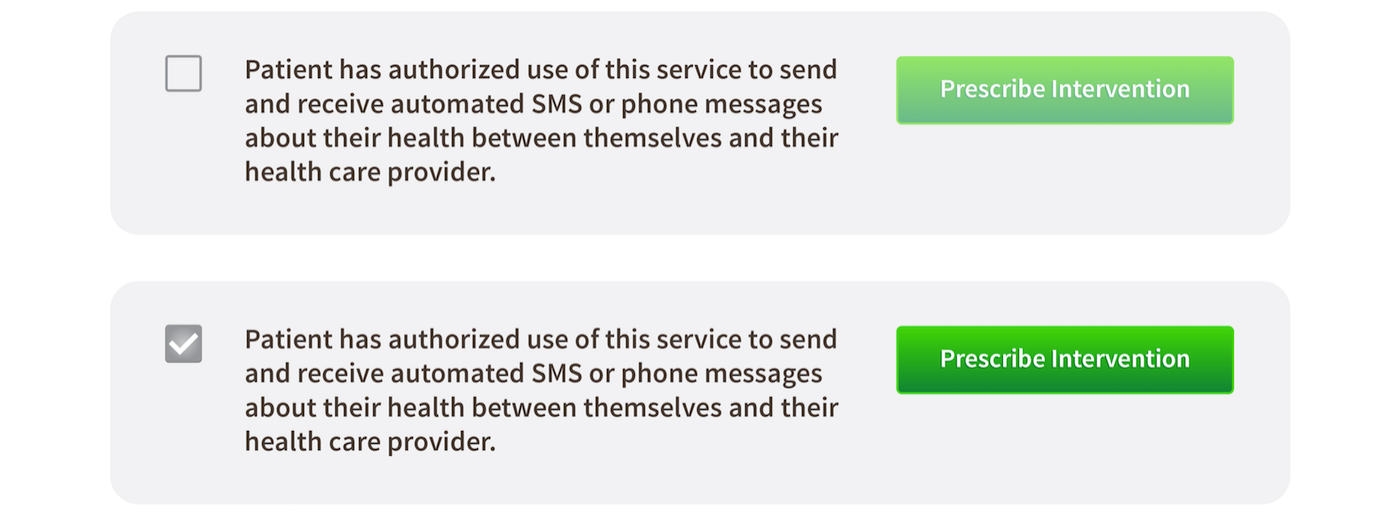
In the unauthorized state, Epharmix will not allow a person to receive the automated message. Once authorized, patients may begin receiving Epharmix automated messages.

The user interface of Epharmix inhibits the sending of messages without a confirmation of consent by the patient as noted in Figure 2 below. This checkbox verification feature reduces the risk of messaging without consent and builds in safeguards for compliance with both HIPAA and TCPA.

Using the methods described above, this process has facilitated the completion of 52,403 phone calls (46%) and text messages (54%). Messages do not contain any identifiers, and this mitigates the risk of a text message or phone call releasing PHI to an incorrect person in cases where the phone number entered into the Epharmix system is incorrect. At the same time, text messages and phone calls are easier for patients to use, fitting more into their activities of daily living as compared with more traditional mailers, fliers, or Web-based portals.

### Patient Engagement

**Figure 3:**
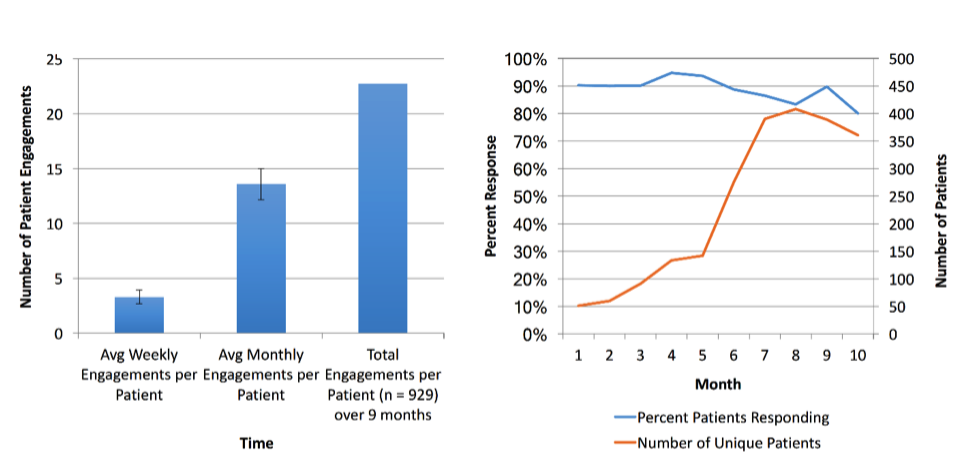
Average number of patient engagements with Epharmix (left) and percentage of patients responding to Epharmix interventions (right).

We find a significant number of engagements, with an average of 13 bidirectional message exchanges with individual patients via text or phone calls per month. Monthly engagement rates have been stable at 80% to 90%, even as the number of patients on Epharmix has steadily risen (Figure 3). Over the entire course, 14.5% of patients explicitly opted out of usage of the service. Although not directly comparable, these engagement rates may suggest higher patient engagement than that seen in portal usage alone in the literature [19,20].

### Satisfaction

**Figure 4:**
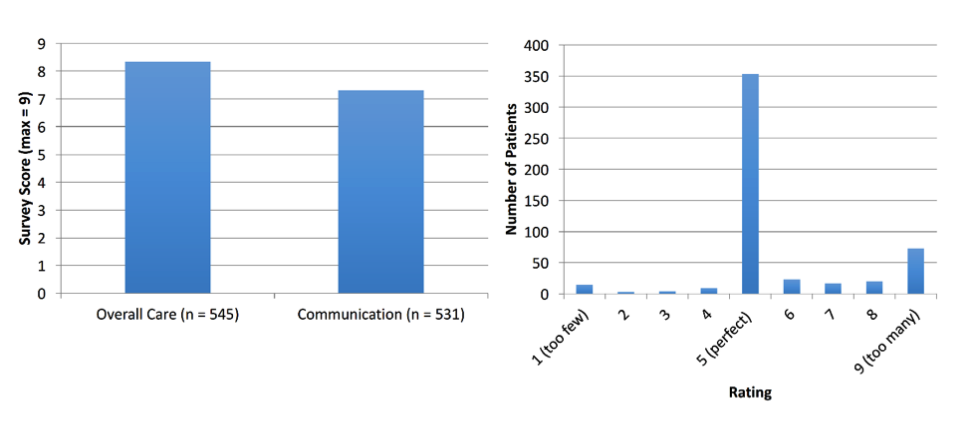
Average patient feedback scores (left) and message frequency feedback ratings (right).

We find that these engagements tend to increase overall satisfaction with patients’ providers (8.4/9), and patients report an increased degree of communication with their provider (7.3/9). In this survey, a response of 5 referred to the same level of satisfaction or communication, 9 represented more, and less than 5 represented less. A majority of patients felt the frequency of messages was about perfect even as the absolute number of engagements in a year with their provider increased from an average of 3 office visits to about 23 times in a year while using Epharmix (Figure 4).

### Clinically Actionable Events

Out of the 52,403 messages sent and received over the course of nine months, there were 1,164 clinically significant events caught, including diabetic hypoglycemia and hyperglycemia, COPD exacerbations, heart failure decompensations, hypertensive crises, wound infections, and breastfeeding complications. These were triggered whenever a patient’s responses crossed any of the thresholds as described in Table 2. Once caught by the Epharmix automated system, proactive care was able to be provided as deemed appropriate by the patient’s specific provider. Accomplishing this degree of proactive care through an alternative method, such as a provider initiated phone bank, would be cost prohibitive and most likely associated with lower patient engagement [27-29].

**Table 2:** Types of clinically significant events identified during the first 18 months of the study.

Intervention	Clinical event
Diabetes	Hypoglycemia (fasting blood glucose <70 mg/dL) Hyperglycemia (fasting blood glucose >400 mg/dL)
Chronic obstructive pulmonary disease	Worsening Dyspnea Fluid overload: increases in weight (>5 lbs over 1 week as compared to baseline)
Congestive heart failure	Hypertensive crisis (blood pressure >180/110 mm Hg) Hypertensive urgency (blood pressure >180/120 mm Hg) Tachycardia (heart rate >100 bpm) Worsening dyspnea, orthopnea, or pedal edema
Hypertension	Hypertensive crisis (blood pressure >180/110 mm Hg) Hypertensive urgency (blood pressure >180/120 mm Hg)
Decolonization	Failure to accept prescribed supplies
Wound	Signs of Infection (worsening pain, drainage, redness, and fever)
Breastfeeding	Patient not exclusively breastfeeding Breastfeeding associated (eg, breast pain, difficulty latching, not producing enough milk, insufficient child weight gain)

## Discussion

### Principal Findings

Building an automated text messaging and phone call system to engage, monitor, and aid patients throughout their care process fundamentally increases engagement. Patients clearly are satisfied with the increased degree of engagement with their providers, and based on our results, find the increased frequency of interactions to be reasonable. The number of clinically significant events identified by this proactive care system emphasizes the utility of distributing accessible electronic messaging systems to patients in traditionally medically underserved areas.

Epharmix provides an example of a solution capable of engaging patients using ubiquitous text messages and phone calls. These lines of communication provide valuable and direct contact approaches, as 80% of cell phone users send and receive text messages [30]. The ability to combine smart algorithmic methods across populations makes this technology much more powerful. Given the high penetration rate of cellphones and landlines among all socioeconomic strata, particularly those with lower incomes, the system enables potentially greater engagement by that otherwise difficult-to-reach patient population.

### Limitations

In focusing on developing a system that uses a widespread infrastructure, landlines and cell phones, the system does remain limited in that it does not reach patients who do not have access to landlines or cell phones. Moreover, it does not take advantage of higher end smartphone features such as video conferencing or using Web links. These are possible future features to add to the system as the underlying smartphone technology becomes more standardized, cheaper, and prevalent in older and lower socioeconomic strata.

This study does not determine whether the high engagement correlates to improved clinical outcomes, and we could not correlate the engagement with satisfaction directly because the data is deidentified and all the clinical conditions are aggregated. Because of the aggregate nature of this study we were unable to assess engagement rates by disease states. We are only observing user statistics and so do not know about nonuser health engagement and use, and finally we lack demographic data to make correlations between age and socioeconomic status and engagement. The series of quality improvement projects and RCTs on which this meta-analysis is based will report whether the increased engagement and early detection of clinically significant events associated with Epharmix will improve underlying clinical outcomes.

### Conclusions

Using accepted means for mitigating risk, we have been able to create a method using ubiquitous and patient-accessible forms of communication—SMS and phone calls—to improve patient engagement, satisfaction, and the capacity for remote monitoring. We find a significantly increased patient engagement rate that has allowed for the identification of numerous clinically significant events that may otherwise have been missed. The use of this monitoring system allows for the early detection and intervention of clinically significant events within the confines of all compliance requirements. In the context of the literature, great progress has been made in developing 1- and 2-way patient-facing portals, mobile phone apps, and even texting [5,11,20,27]. Developing a two way automated SMS system across multiple disease states is still a challenge for both technical, and security based reasons. The system described enables the use of automated SMS and phone calls to help increase patient engagement feasibly and increase two way feedback loops across multiple disease states. Future directions include analysis per specific disease state and randomized controlled trials to study the impact.

## Abbreviations

COPD: chronic obstructive pulmonary disease
EMR: electronic medical record
FCC: Federal Communications Commission
HITECH: Health Information Technology for Economic and Clinical Health Act
HIPAA: Health Insurance Portability and Accountability Act
PHI: protected health information
RCT: randomized controlled trial
SMS: short message service
TCPA: Telephone Consumer Protection Act
